# Supplementary material for: Myoelectric digit action decoding with multi-output, multi-class classification: an offline analysis
Source: Sci Rep. 2020 Oct 9;10:16872. doi: 10.1038/s41598-020-72574-7 (PMC7547112; doi:10.1038/s41598-020-72574-7)
Supplement: Supplementary file 1 — Supplementary Information. [file 41598_2020_72574_MOESM1_ESM.pdf]

Supplementary information: Myoelectric digit action decoding with multi-output, multi-class classification: an offline analysis

Agamemnon Krasoulis[1,*] **and Kianoush Nazarpour**[1,2]

[1]School of Engineering, Newcastle University, Newcastle upon Tyne, NE1 7RU, UK
[2]School of Informatics, University of Edinburgh, Edinburgh, EH8 9QD, UK
[*]Agamemnon.Krasoulis@newcastle.ac.uk; Kianoush.Nazarpour@ed.ac.uk

Supplementary figures

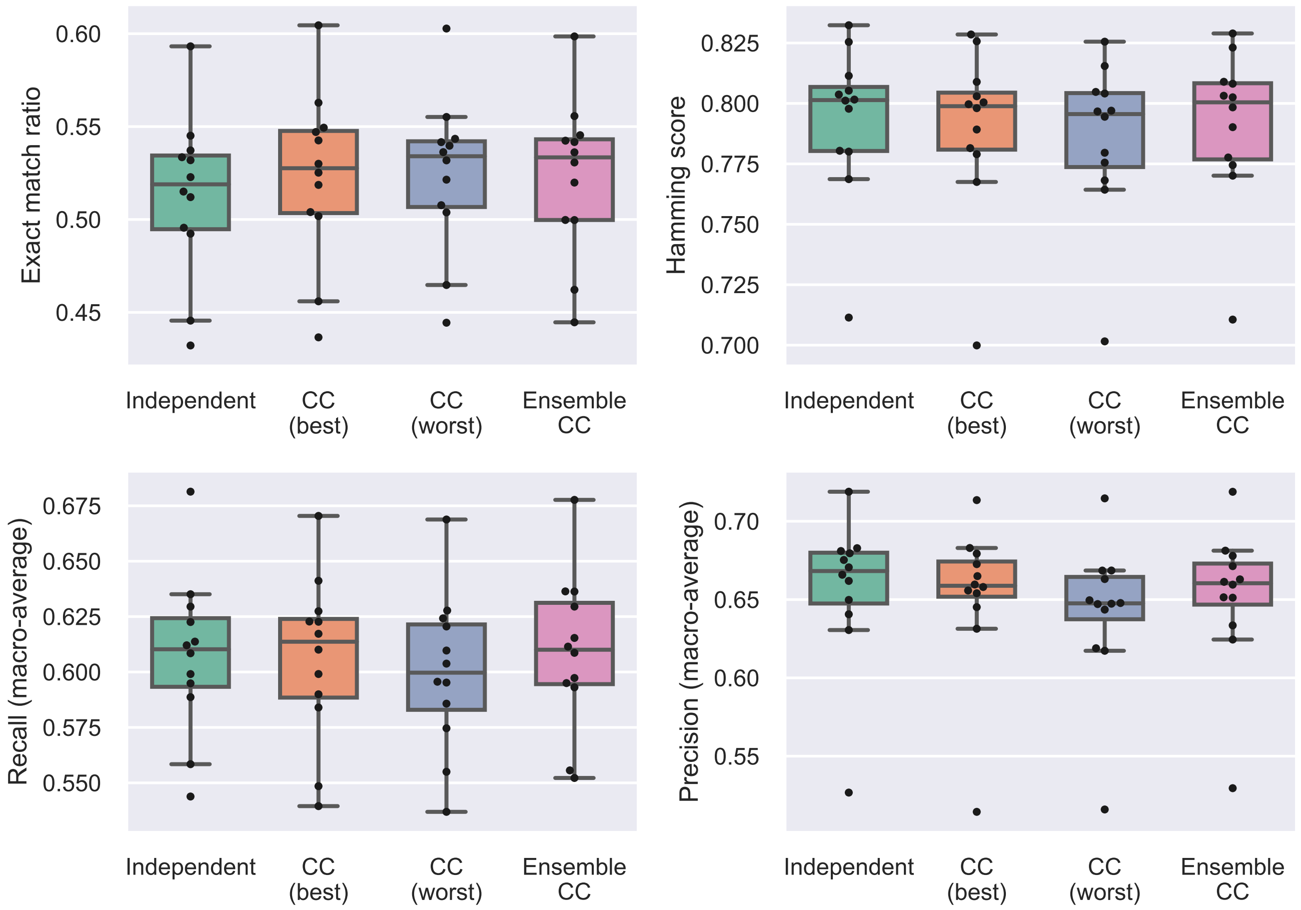

Figure S1. Classifier chain comparison using additional performance measures.

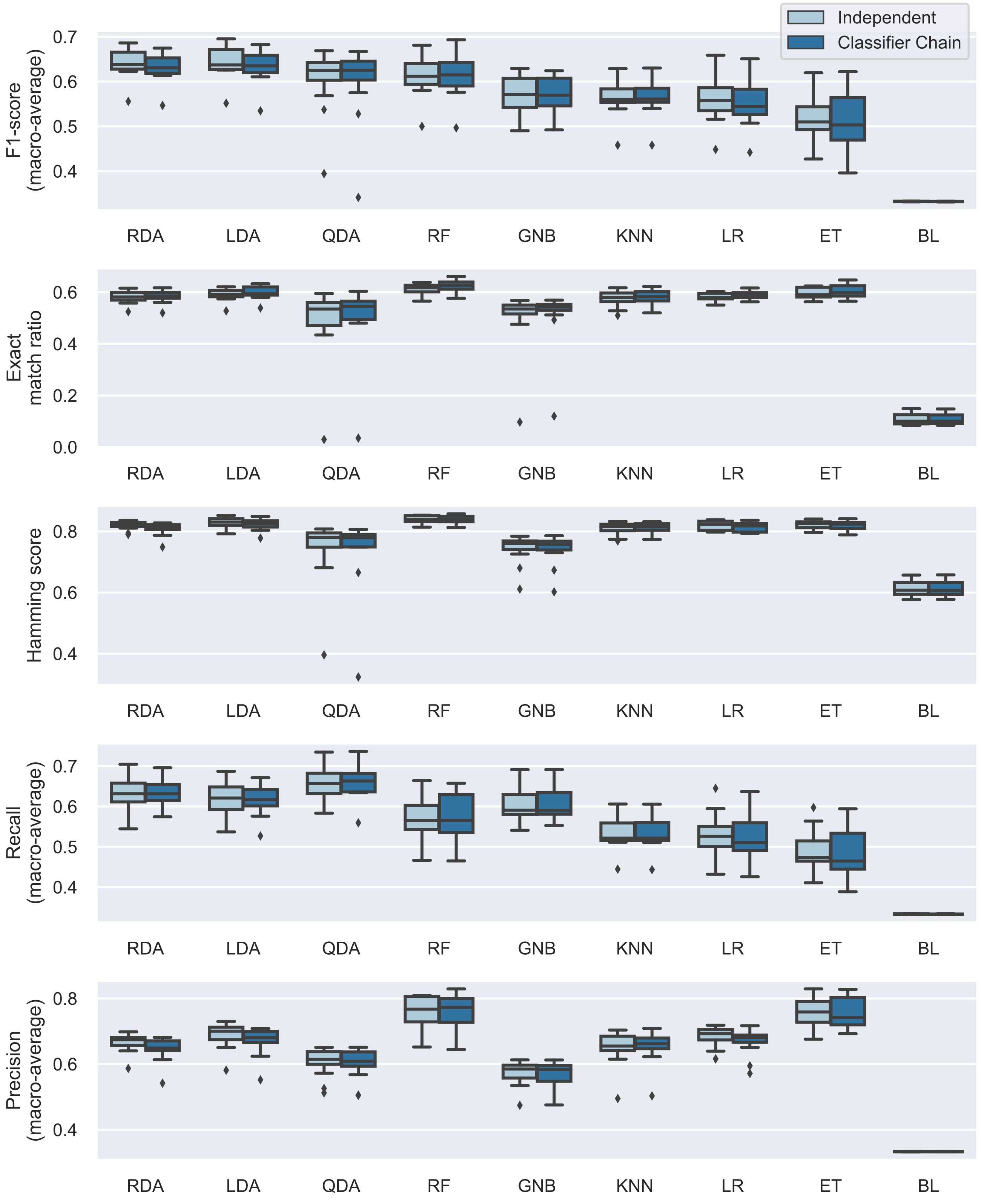

Figure S2. Multi-output independent classifier comparison using additional performance measures. Algorithms are presented in order of decreasing median macro-average F1-score. Diamonds indicate outliers.

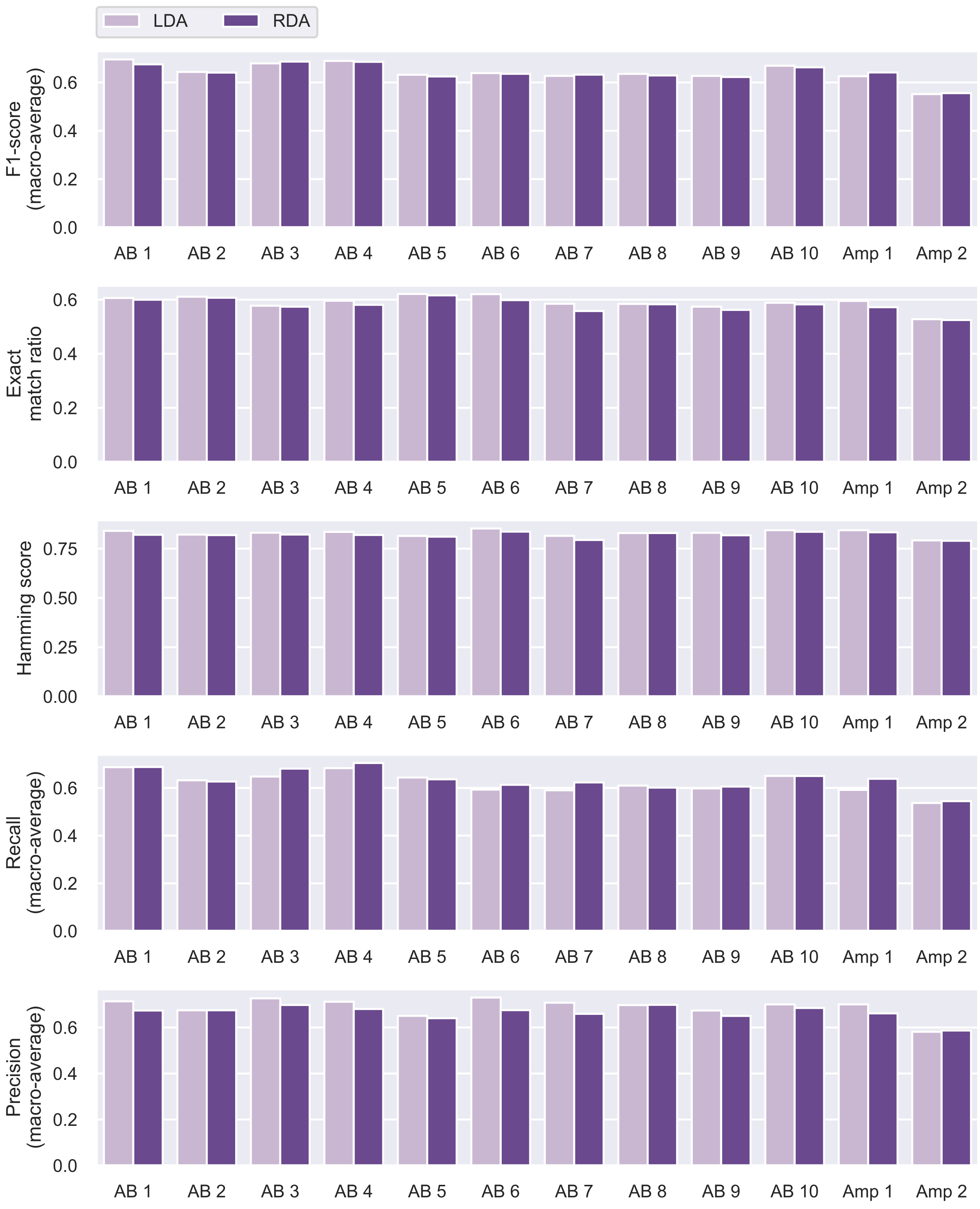

Figure S3. Multi-output independent classification performance with best-performing algorithms for individual participants. AB, able-bodied; Amp, amputee.

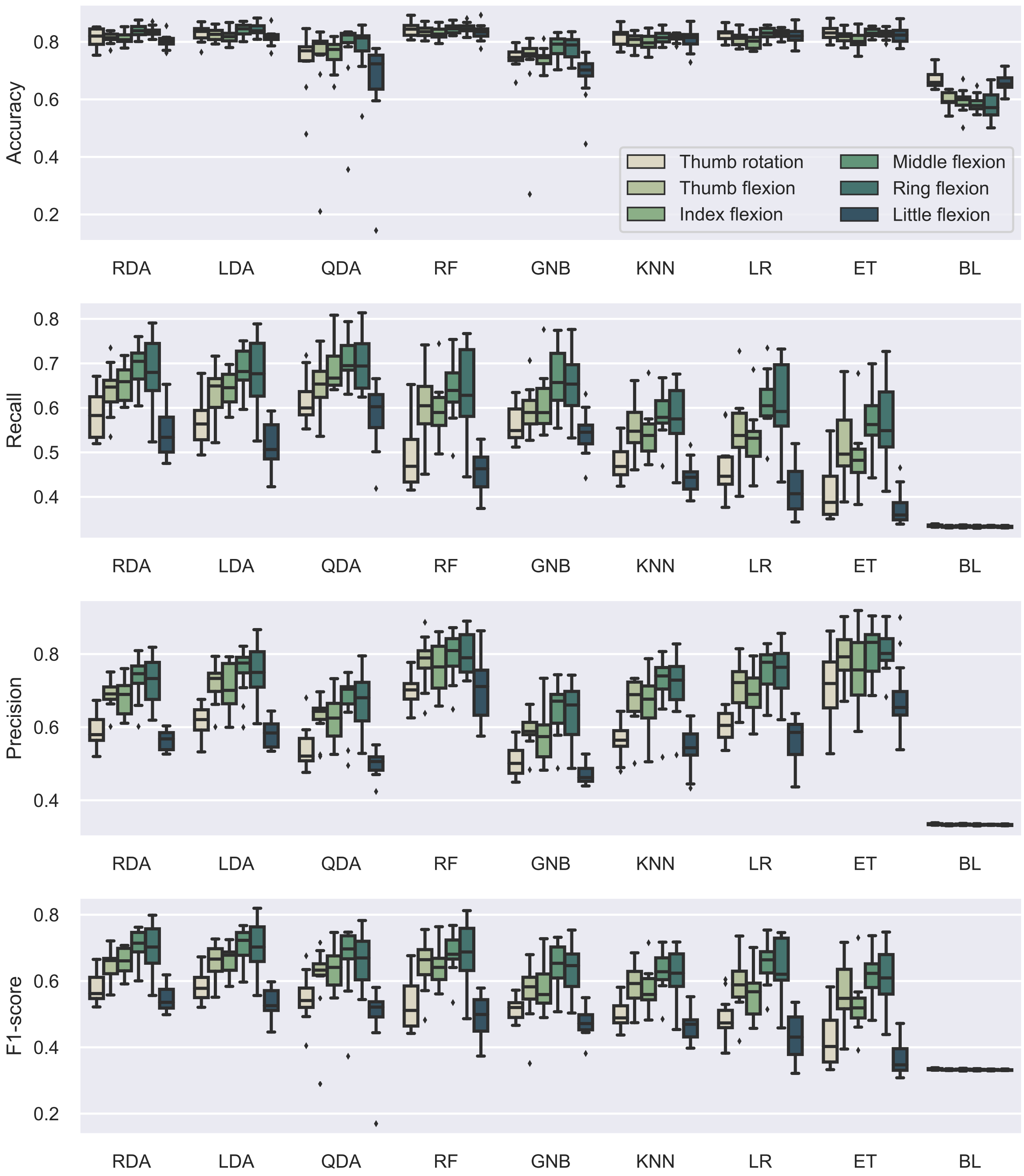

Figure S4. Multi-output independent classifier comparison for individual DOFs using additional performance measures. Algorithms are presented in order of decreasing median macro-average F1-score.

Supplementary table

Table S1. Classification benchmark. Abbreviations, hyper-parameters, and search ranges.

**Classifier**	**Abbrv.**	**Hyper-parameter**	**Random search range**
Baseline	BL		
Logistic regression	LR	Regularisation parameter	logspace{min=10^{-5}, max=10^{5}, n=100}
Linear discriminant analysis	LDA		
Quadratic discriminant analysis	QDA		
Regularised discriminant analysis	RDA	Regularisation parameter α	linspace{min=0, max=1, n=101}
		Regularisation parameter γ	linspace{min=0, max=0.2, n=21}
Gaussian Naive Bayes	GNB		
K-nearest neighbours	KNN	Number of neighbours	linspace{min=1, max=50, n=51}
		Weights	{uniform, distance}
Random forests	RF	Number of estimators	{10, 20, 50, 100, 200, 500}
		Maximum number of features	{auto, sqrt, log2}
		Maximum depth	linspace{min=1, max=10, n=11}
		Criterion	{gini, entropy}
Extra trees	ET	Number of estimators	{10, 20, 50, 100, 200, 500}
		Maximum number of features	{auto, sqrt, log2}
		Maximum depth	linspace{min=1, max=10, n=11}
		Criterion	{gini, entropy}
